# Characterization of multidrug-resistant ST363-KL151 *Klebsiella variicola* clinical isolates harboring *bla*_NDM−9_ from Eastern China

**DOI:** 10.1186/s12866-026-05170-w

**Published:** 2026-05-19

**Authors:** Ling Wang, Huiming Li, Si Xu, Zhiyou Xiao, Derong Xu, Yiqi Wan

**Affiliations:** 1 Department of Clinical Laboratory, Medical Center of Burn plastic and wound repair, The First Affiliated Hospital, Jiangxi Medical College, Nanchang University, Nanchang, 330006 China; 2 Jiangxi Institute of Translational Medicine, The First Affiliated Hospital, Jiangxi Medical College, Nanchang University, Nanchang, Jiangxi 330000 China; 3 Jiangxi Key Laboratory of Drug Target Discovery and Validation, Jiangxi Medical College, Nanchang University, Nanchang, Jiangxi 330000 China

**Keywords:** *Klebsiella variicola*, ST363-KL151, NDM-9, resistance, virulence

## Abstract

**Background:**

*Klebsiella variicola* is an emerging multidrug‑resistant opportunistic pathogen that is often misidentified as *K. pneumoniae* in clinical laboratories. Here, we investigated four NDM‑9-producing *K. variicola* ST363‑KL151 isolates from patients treated at a tertiary-care centre in China.

**Methods:**

Whole-genome sequencing and genomic analyses were performed to confirm species identity, define molecular characteristics, and identify resistance determinants. Core-genome SNP analysis was used to assess phylogenetic relatedness to international strains. Carbapenem-resistance transferability was evaluated by conjugation to *E. coli*. Biofilm formation, epithelial-cell invasion/cytotoxicity, serum bactericidal resistance, and virulence in a *Galleria mellonella* infection model were assessed.

**Results:**

Antimicrobial susceptibility testing revealed a broad resistance profile encompassing β‑lactams (including carbapenems), aminoglycosides, fluoroquinolones, and fosfomycin, while retaining susceptibility to tigecycline. One isolate demonstrated elevated polymyxin resistance with an MIC of 64 mg/L, mediated by *mgrB* truncation (p.Gln30*). Whole‑genome sequencing identified a IncHI2A plasmid carrying a unique IS*26*‑flanked *bla*_NDM‑9_ composite transposon adjacent to a mercury resistance operon, and conjugation assays confirmed transferable carbapenem resistance. Phylogenetic analysis placed the ST363-KL151 isolates in a monophyletic clade with a Norwegian strain collected in 2015. Although hypervirulence plasmids and classical virulence loci were absent, the isolates showed substantial biofilm formation and serum resistance, but only moderate cytotoxicity in epithelial cells and limited lethality in *Galleria mellonella* compared with the hypervirulent control NTUH-K2044.

**Conclusions:**

These findings identify NDM‑9‑producing *K. variicola* ST363‑KL151 as a multidrug‑resistant lineage, emphasizing the need for accurate species identification and genome‑based surveillance to prevent its further clinical spread.

**Supplementary Information:**

The online version contains supplementary material available at 10.1186/s12866-026-05170-w.

## Introduction

Carbapenem-resistant Enterobacterales have spread worldwide and represent a major public health threat because they limit treatment options and complicate infection control [1]. *Klebsiella variicola*, a member of the *Klebsiella pneumoniae* species complex, is increasingly recognized as an opportunistic pathogen, although its clinical significance remains underestimated [2, 3]. It can cause bloodstream, respiratory, urinary tract, and wound infections [4–6]. Although first described as a plant endophyte [7], *K. variicola* is now known to circulate widely among human, animal, and environmental reservoirs [2]. However, its close relatedness to *K. pneumoniae* often leads to misidentification by routine methods such as MALDI-TOF MS and biochemical systems [3, 8], obscuring its true epidemiology.

Carbapenem resistance in *K. variicola* is increasingly reported and is mainly driven by horizontal acquisition of carbapenemase genes [9, 10]. Among these, New Delhi metallo-β-lactamase (NDM) is particularly concerning because it confers resistance to nearly all β-lactams and is often linked to multidrug resistance and plasmid-mediated dissemination [11–14]. The *bla*_NDM‑9_ variant, first identified in China, has since been detected in multiple Enterobacterales species and regions [15, 16]. However, *bla*_NDM‑9_-producing *K. variicola* remains rarely described, and its genetic context and dissemination potential remain unclear.

Although *K. variicola* generally lacks the classical hypervirulence determinants found in hypervirulent *K. pneumoniae*, including *rmpA*/*rmpA2* and siderophore loci [17, 18], it retains important pathogenic traits, including capsule production, lipopolysaccharide synthesis, and fimbrial adhesins [17]. Experimental and clinical evidence indicates that even non-hypervirulent strains can cause severe infection, especially in immunocompromised or critically ill patients [4, 6, 19]. Therefore, elucidating the virulence and resistance features of multidrug‑resistant *K. variicola* is essential for understanding its clinical impact and guiding appropriate therapeutic and infection control strategies.

In this study, we analysed four ST363 *K. variicola* isolates of carrying the *bla*_NDM‑9_ gene. Comparative genomic analyses were performed to define the molecular characteristics and plasmid architecture of this lineage and to determine its relatedness to global *K. variicola* strains. We also assessed key virulence-associated phenotypes to evaluate the pathogenic potential of this emerging lineage.

## Materials and methods

### Isolate identification and culture

Four non-duplicate *K. variicola* isolates were recovered from clinical specimens submitted to the clinical microbiology laboratory of the First Affiliated Hospital of Nanchang University between January and June 2025. Identification of the isolates was initially performed by MALDI-TOF MS, and colonies with a score ≥ 1.9 were considered as reliable species identification. The reference *K. pneumoniae* strain NTUH-K2044 was obtained from National Taiwan University Hospital and included as a reference strain [20].

### Antimicrobial susceptibility testing

Antimicrobial susceptibility was determined by broth microdilution according to CLSI M100, 33rd edition. MICs of antimicrobial agents were measured in cation-adjusted Mueller-Hinton broth and incubated at 37 °C for 18–24 h. Cefiderocol MICs were determined in iron-depleted CAMHB as recommended by CLSI [21]. Results were interpreted using CLSI breakpoints, except for tigecycline and polymyxin B, which were interpreted according to EUCAST criteria. *Escherichia coli* ATCC 25,922 and *Pseudomonas aeruginosa* ATCC 27,853 served as quality control strains.

### Whole Genome Sequencing (WGS) and analysis

WGS of all four ST363-KL151 isolates was performed on the Illumina NovaSeq platform at a depth of at least 100×. Reads were trimmed and quality-filtered with fastp v0.20.1 [22], assembled using SPAdes v3.15.0 [23], and assessed with QUAST v5.0.2 [24]. Sequence type, capsular type, and O antigen serotype were determined using the PubMLST *Klebsiella* scheme and Kleborate v2.3.1 [25]. Antimicrobial resistance and virulence genes were identified using ResFinder and VFDB [26, 27], with minimum identity and coverage thresholds of 80%. For KV24, additional long-read sequencing was performed using the PacBio platform. Hybrid assembly was generated with Unicycler v0.4.8 [28], and the complete genome was annotated using Prokka v1.14.6 [29].

### Construction of the *mgrB* mutant

The chromosomal *mgrB* mutation was reversed using the CRISPR-Cas9 editing system consisting of pCasKP-apr and pSGKP-spe, as previously described [30]. The editing procedure involved two sequential homologous recombination steps. First, linear donor DNA and a spacer-containing pSGKP-spe plasmid were co-electroporated into recipient strains carrying pCasKP-apr following L-arabinose induction, resulting in deletion of the *mgrB* region surrounding the target site. After partial gene deletion, the pSGKp plasmid was cured by culturing the bacteria on LB agar containing apramycin and 5% sucrose at 30 °C. In the second step, the wild-type *mgrB* sequence was reintroduced into the chromosome using a repair template together with a pSGKP plasmid targeting the deleted locus, thereby reverting the c.88 C > T mutation. After curing of both plasmids, colonies were screened by PCR, and successful reversion was confirmed by Sanger sequencing. The colistin MICs of the resulting revertant strains were determined by broth microdilution. All primers used in this study are listed in Table S1.

### Phylogenetic analysis

A total of 156 *K. variicola* genomes were retrieved from the NCBI RefSeq database and analyzed together with the four isolates sequenced in this study. SNP calling was performed using Snippy v4.6.0 (https://github.com/tseemann/snippy) with *K. variicola* ATCC BAA-830 (RefSeq accession GCF_020525545.1) as the reference genome. Pairwise SNP distances among all isolates were calculated using SNP-dists v0.8.2 (https://github.com/tseemann/snp-dists). Recombinant regions were identified and masked using Gubbins v3.2.1 [31], and the filtered alignment was used to infer a maximum-likelihood phylogeny with FastTree v2.1 [32]. The tree was visualized and annotated using iTOL [33].

### Conjugation assay

Conjugation assays were performed to evaluate horizontal transfer of resistance plasmids from the four ST363-KL151 *K. variicola* isolates. Each clinical isolate (KV24, KV63, KV79, and KV102) was used as a donor, and sodium azide-resistant *E. coli* J53 served as the recipient. Mid-log-phase donor and recipient cultures were mixed at a 1:1 ratio, spotted onto LB agar, and incubated overnight at 37 °C. Cells were then recovered and plated on selective agar containing sodium azide (100 µg/mL) and meropenem (2 µg/mL). Conjugation frequency was calculated as transconjugants per donor cell. Presumptive transconjugants were identified by MALDI-TOF MS, and plasmid transfer was confirmed by PCR detection of *bla*_NDM−9_, *bla*_TEM−1B_, and *aadA2*. Primer sequences are listed in Table S1.

### Biofilm formation assay

Biofilm formation was quantified in 96-well polystyrene plates using a crystal violet assay [34]. Overnight cultures grown in LB broth at 37 °C were diluted 1:100 in fresh LB, and 200 µL aliquots were inoculated into sterile microplates. After static incubation at 37 °C for 24 h, wells were washed with phosphate-buffered saline, fixed with methanol, stained with 0.1% crystal violet, and destained with 33% acetic acid. Absorbance was measured at 600 nm using a microplate reader. Uninoculated LB served as the negative control, and NTUH-K2044 and ATCC 700,603 were included as reference strains. Each strain was tested in triplicate, and mean OD_600_ value was calculated.

### Bacterial invasion

Human lung adenocarcinoma epithelial cells (A549; American Type Culture Collection, ATCC, CCL-185) were maintained in DMEM containing 10% FBS and 1% penicillin–streptomycin under standard conditions (37 °C, 5% CO₂). For invasion assays, cells were seeded in 24-well plates at 1 × 10^5^ cells/well and grown to confluence. Overnight bacterial cultures were adjusted to OD_600_ = 0.5 in antibiotic-free DMEM and used to infect A549 monolayers at a multiplicity of infection (MOI) of 10:1. After 30 min of incubation, monolayers were washed with PBS and incubated for 1 h in DMEM containing gentamicin (100 µg/mL) to kill extracellular bacteria. Cells were then washed, lysed with 0.1% Triton X-100, and intracellular bacteria were quantified by plating serial dilutions on LB agar. Each assay was performed in triplicate.

### Cytotoxicity assay

A549 cells were cultured as described above and maintained in antibiotic-free DMEM before infection. Overnight bacterial cultures were washed, resuspended in PBS, and added to A549 monolayers at an MOI of 100:1. After 6 h of incubation at 37 °C in 5% CO₂, culture supernatants were collected and clarified by centrifugation (3,000×g, 5 min, 4 °C). Lactate dehydrogenase (LDH) release was measured using a commercial kit (Solarbio, BC0685), with absorbance read at 490 nm. Cytotoxicity was expressed relative to the maximum LDH release from cells lysed with 1% Triton X-100, with uninfected cells as the baseline control. Each condition was tested in triplicate in three independent experiments.

### Serum killing assay

Serum bactericidal activity was assessed using normal human serum prepared from fresh blood collected from healthy volunteers without recent antibiotic exposure. Overnight bacterial cultures were washed, resuspended in PBS, and adjusted to approximately 1 × 10⁶ CFU/mL. Aliquots of bacterial suspension (100 µL) were mixed with serum (500 µL) and incubated at 37 °C. Samples were collected at 0, 60, 120, and 180 min, serially diluted in PBS, and plated on LB agar for colony counting after overnight incubation. All assays were performed in triplicate.

### *Galleria mellonella* infection assay

*Galleria mellonella* larvae (250–300 mg) were randomly assigned to treatment groups and injected with 10 µL of bacterial suspension containing 1 × 10⁶ CFU using a Hamilton syringe. Injections were administered via the last left proleg. Control larvae received sterile 0.9% sodium chloride. After inoculation, larvae were incubated at 37 °C in the dark, and survival was monitored every 12 h for 72 h. Larvae showing no response to mechanical stimulation and complete melanization were considered dead. Each group contained 10 larvae, and experiments were performed in triplicate.

### Statistical analysis

Data analysis was conducted in GraphPad Prism 9.0 (GraphPad Software, San Diego, CA, USA). Differences among groups were evaluated by one‑way ANOVA with Tukey’s multiple‑comparisons test as needed. Results are reported as mean ± SD, and error bars indicate SD. Two‑sided *P* < 0.05 was considered statistically significant.

## Results

### Clinical characteristics of patients infected with NDM‑9‑Producing *K. variicola*

Four non-duplicate NDM-9-producing *K. variicola* isolates (KV24, KV63, KV79, and KV102) were recovered from four inpatients in different departments between January and June 2025 (Table 1). Two isolates (KV24 and KV63) were obtained from blood cultures, whereas KV79 and KV102 were recovered from bronchoalveolar lavage fluid and sputum, respectively. All patients had severe underlying comorbidities and had undergone at least one invasive procedure before culture positivity. Polymicrobial infection was documented in two cases, and *Pseudomonas aeruginosa* was co-isolated from two patients. Before isolation of *K. variicola*, all patients had received broad-spectrum antibiotics, most commonly a carbapenem combined with a β-lactam/β-lactamase inhibitor. After multidrug resistance was identified, antimicrobial therapy was adjusted according to susceptibility testing. Despite these interventions, clinical outcomes were poor: two patients died, one patient was discharged for palliative care, and one recovered and was discharged after polymyxin B-based therapy.

**Table 1:** Clinical features of patients infected with NDM‑9‑producing *K. variicola*

Case	Isolate	Age/Sex	Specimen	Department	Diagnosis	Underlying disease	Invasive procedures	Co-isolated pathogen
1	KV24	56 / M	Blood	Nephrology Ward	Sepsis secondary to urinary infection	Type 2 diabetes, hypertension	Central venous catheterization	None detected
2	KV63	78 / F	Blood	Intensive Care Unit	Hospital-acquired pneumonia with bacteremia	COPD, heart failure	Endotracheal intubation, mechanical ventilation	None detected
3	KV79	71 / M	Bronchoalveolar lavage fluid	Intensive Care Unit	Ventilator-associated pneumonia	Chronic kidney disease, diabetes	Mechanical ventilation, hemodialysis	*Pseudomonas aeruginosa*
4	KV102	63 / F	Sputum	Oncology Ward	Bacteremia with pulmonary infection	Hypertension, gastric carcinoma	Central venous catheterization	*Pseudomonas aeruginosa*

### Identification of ST363‑KL151 *K. variicola* isolates

Although initially identified as *K. pneumoniae* by MALDI-TOF MS, all four isolates were confirmed by whole-genome sequencing as *K. variicola*. Draft genome assemblies were of high quality, with sizes ranging from 5.72 to 5.82 Mb. MLST analysis placed all isolates in ST363 (*gapA*16, *infB*18, *mdh*21, *pgi*27, *phoE*53, *rpoB*22, and *tonB*67). Capsule typing revealed a uniform KL151 K locus and O antigen type O5, corresponding to wzi allele 427. Kleborate analysis assigned virulence scores of 0 to all isolates, and no virulence plasmids or hypervirulence-associated loci, including *rmpA*, *rmpA2*, or *rmpADC*, were detected. Likewise, major siderophore loci such as yersiniabactin, aerobactin, colibactin, and salmochelin were absent, consistent with a non-hypervirulent genotype. All isolates carried several chromosomally encoded virulence-associated genes, including *entA*, *entB*, and *fepC* involved in the enterobactin siderophore system, *ompA* encoding an outer membrane protein, and *ykgK* and *yagVWXYZ* associated with adhesion and fimbrial or pilus biosynthesis.

### Resistance characteristics of ST363‑KL151 *K. variicola* isolates

The resistance scores ranged from 2 to 3, spanning eight to nine antimicrobial classes. Common resistance determinants included *bla*_NDM‑9_, *bla*_TEM‑1B_, *bla*_LEN‑24_, *fosA3*, *dfrA12*, *sul1*, *sul3*, and several aminoglycoside‑modifying enzymes (*aac(3)‑IV*, *aadA2*, and *aph(3′)‑Ia*). In addition, genes conferring resistance to macrolides and chloramphenicol (*lnuF*, *mphA*, and *cmlA1*) were detected in all four isolates. Notably, the KV63 isolate contained a premature stop codon in the *mgrB* gene (c.88 C > T; p.Gln30*). Antimicrobial susceptibility testing demonstrated that all four *K. variicola* ST363‑KL151 isolates exhibited a multidrug‑resistant phenotype (Table 2). All were resistant to carbapenems, aztreonam, cefiderocol, aminoglycosides, and levofloxacin, but remained susceptible to tigecycline. Notably, reduced susceptibility to polymyxins was confined to isolate KV63, which displayed a markedly elevated polymyxin B MIC of 64 mg/L, while KV24, KV79, and KV102 remained susceptible. To confirm the role of the *mgrB* mutation in polymyxin B resistance, the *mgrB* nonsense mutation (c.88 C > T; p.Gln30*) in KV63 was reverted to the wild-type allele using CRISPR-Cas9-mediated allelic repair. The resulting isogenic revertant showed a substantial decrease in the polymyxin B MIC, from 64 mg/L to 2 mg/L.

**Table 2:** Antimicrobial susceptibility profiles of NDM‑9‑producing ST363‑KL151 K. *variicola* isolates

Antimicrobial agent	KV24	KV63	KV79	KV102
Aztreonam	64 (R)	64 (R)	64 (R)	64 (R)
Amikacin	64 (R)	64 (R)	64 (R)	64 (R)
Ampicillin-sulbactam	64/16 (R)	64/16 (R)	64/16 (R)	64/16 (R)
Piperacillin-tazobactam	32/4 (R)	64/4 (R)	64/4 (R)	32/4 (R)
Cefiderocol	16 (R)	16 (R)	16 (R)	16 (R)
Cefuroxime	64 (R)	64 (R)	64 (R)	64 (R)
Ceftazidime	> 128 (R)	> 128 (R)	> 128 (R)	> 128 (R)
Ceftazidime-avibactam	128/4 (R)	128/4 (R)	128/4 (R)	128/4 (R)
Imipenem	128 (R)	128 (R)	128 (R)	128 (R)
Meropenem	64 (R)	64 (R)	64 (R)	64 (R)
Gentamicin	16(R)	16 (R)	16(R)	16 (R)
Levofloxacin	16 (R)	16 (R)	16 (R)	16 (R)
Tigecycline	1 (S)	1 (S)	1 (S)	1 (S)
Polymyxin B	1 (S)	64 (R)	1 (S)	1 (S)

Abbreviations: *MICs* are expressed in mg/L. *R* Resistant, *I* Intermediate, *S* Sensitive

### Genomic and plasmid characteristics of ST363‑KL151 *K. variicola* KV24

**Fig. 1:**
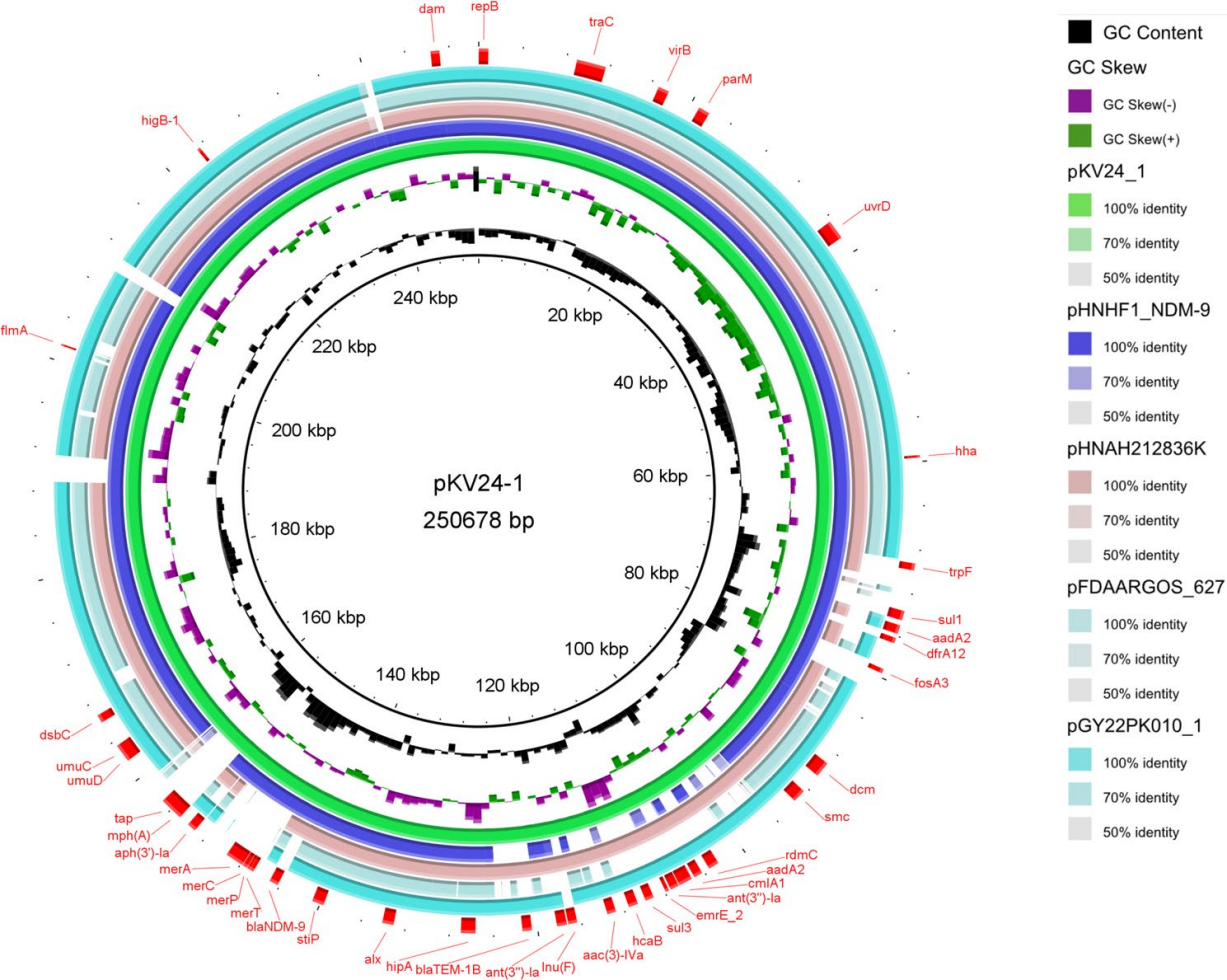
Comparative genomic analysis of plasmid pKV24-1 and related multidrug-resistant plasmids. Comparison of pKV24-1 with four closely related plasmids from different Enterobacterales species: *K. aerogenes* pHNHF1_NDM-9 (CP047668.1), *K. pneumoniae* pHNAH212836K (CP104628.1), *K. variicola* pFDAARGOS_627 (CP044051.1), and *K. aerogenes* pGY22PK010_1 (CP116610.1)

Because the four ST363-KL151 isolates were highly similar in their genomic and antimicrobial resistance profiles, KV24 was selected for long-read sequencing to obtain a complete genome. The completed genome comprised a chromosome and two plasmids, designated pKV24-1 and pKV24-2 (Table 3). Intrinsic resistance determinants, including *bla*_LEN‑24_, *oqxA*, and *oqxB*, were located on the chromosome, whereas most acquired resistance genes were carried by the large IncHI2A plasmid pKV24-1. The smaller plasmid, pKV24-2, did not harbor resistance genes. Comparative genomic analysis showed that plasmid pKV24‑1 exhibited strong sequence homology with previously described multidrug‑resistant plasmids within the Enterobacterales at the whole-plasmid level (Fig. 1). The closest related plasmids were *K. aerogenes* pHNHF1_NDM‑9 (90% query coverage, 99.98% identity; GenBank CP047668.1), *K. pneumoniae* pHNAH212836K (91% coverage, 100% identity; CP104628.1), *K. variicola* pFDAARGOS_627 (77% coverage, 98.62% identity; CP044051.1), and *K. aerogenes* pGY22PK010_1 (89% coverage, 100% identity; CP116610.1).

**Table 3:** Genomic and plasmid features of ST363‑KL151 *K. variicola* KV24

Characteristic	KV24		
	Chromosome	pKV24-1	pKV24-2
Size (bp)	5,168,234	250,678	118,613
GC content (%)	58.05	47.03	50.35
Incompatibility group	/	IncHI2A	IncFIB(K)
Resistance genes	*bla*_LEN24_, *oqxA*,* oqxB*	*aac(3)-IVa*,* aadA2*,* ant(3’’)-Ia*,* aph(3’)-Ia*,* bla*_NDM−9_, *bla*_TEM−1B_, *cmlA1*,* dfrA12*,* fosA3*,* lnu(F)*,* mph(A)*,* sul1*,* sul3*	/
Virulence genes	*entA*, *entB*, *fepC*, *ompA*, *ykgK*, *yagV*, *yagW*, *yagX*, *yagY*, *yagZ*	/	/

**Fig. 2:**
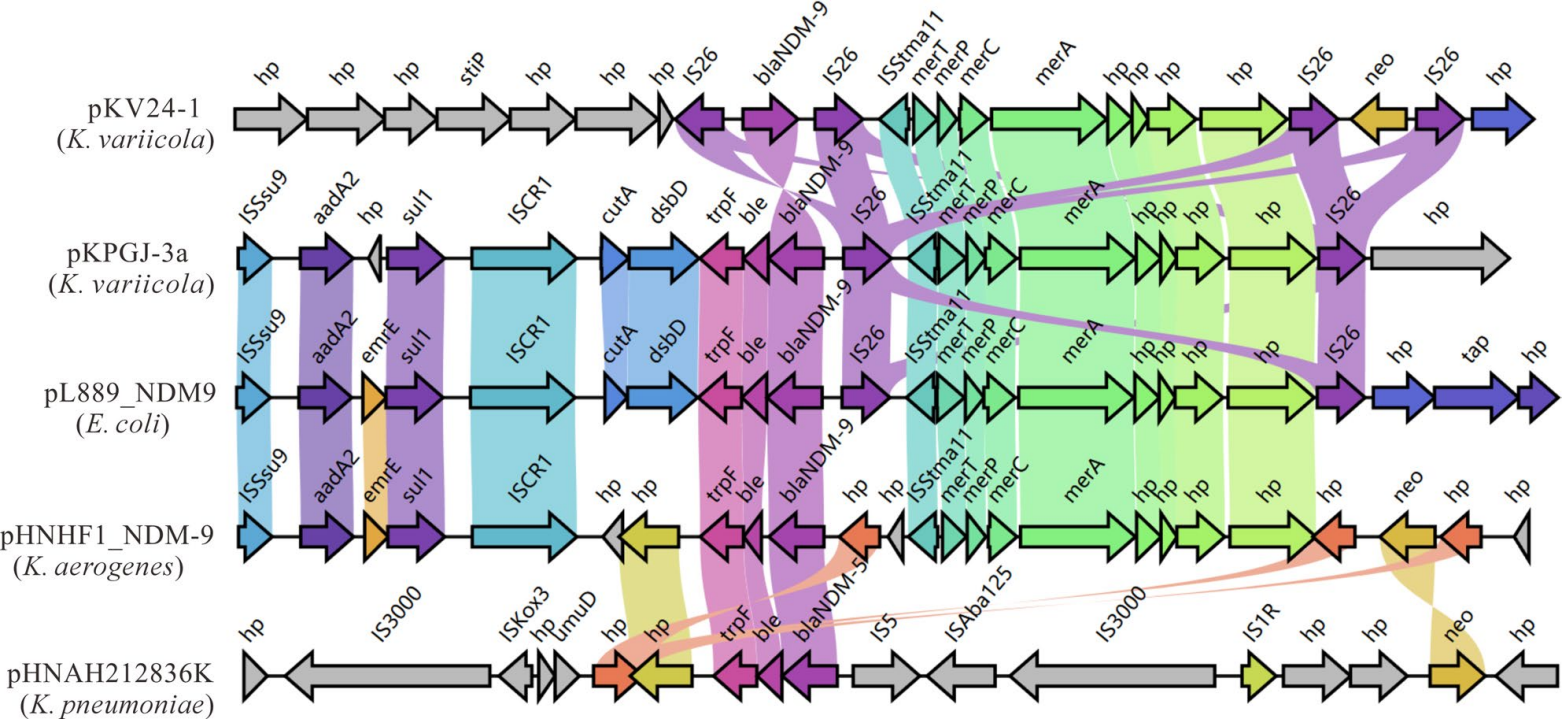
Linear alignment of the *bla*_NDM‑9_ gene cluster from pKV24-1, alongside reference plasmids pKPGJ-3a (*K. variicola*, CP017285.1), pL889_NDM9 (*E. coli*, MZ062604.1), pHNHF1_NDM-9 (*K. aerogenes*, CP047668.1), and pHNAH212836K (*K. pneumoniae*, CP104628.1)

Further analysis of the *bla*_NDM‑9_ region revealed a distinctive genetic organization (Fig. 2). In pKV24‑1, *bla*_NDM‑9_ was situated within a composite transposon bracketed by two copies of IS*26*, forming the arrangement IS*26*–*bla*_NDM‑9_–IS*26*. Upstream of this structure were several genes encoding hypothetical proteins, while downstream lay a complete mercury resistance operon consisting of *merT*, *merP*, *merC*, *merA*, and *merR*. For comparison, *bla*_NDM‑9_ contexts in *K. variicola* plasmid pKPGJ‑3a (CP017285.1) and *E. coli* plasmid pL889_NDM9 (MZ062604.1) displayed a different genetic configuration. Those plasmids carried a module arranged as IS*Ssu9*–*aadA2*–*hp*–*sul1*–*ISCR1*–*cutA*–*dsbD*–*trpF*–*ble*–*bla*_NDM‑9_–IS*26*, including adjacent aminoglycoside and sulfonamide resistance genes (*aadA2* and *sul1*) together with *ISCR1* and *cutA*–*dsbD* segments adjacent to *bla*_NDM‑9_. Conjugation assays using sodium azide-resistant *E. coli* J53 as the recipient demonstrated successful transfer of the IncHI2A plasmid from all four isolates, although transfer frequencies varied (Table 4). The plasmids from KV24, KV79, and KV102 transferred at frequencies of 3.2 × 10⁻^6^ to 1.1 × 10⁻^5^ per donor cell, whereas transfer from KV63 was approximately 10-fold lower.

**Table 4:** Conjugative transfer frequencies of IncHI2A plasmids from ST363‑KL151 Klebsiella variicola isolates

plasmid	Representative resistance genes	No. of independent determinations	Conjugation frequency (Mean)	Range
pKV24-1	*bla*_NDM−9_, *bla*_TEM−1B_, *aadA2*, *cmlA1*, *sul1*	3	1.1 × 10⁻^5^	(8.6 × 10⁻^6^– 1.4 × 10⁻^5^)
pKV63-NDM-9	*bla*_NDM−9_, *bla*_TEM−1B_, *aadA2*, *cmlA1*, *sul1*	3	2.9 × 10⁻^7^	(2.0 × 10⁻^7^ – 3.3 × 10⁻^7^)
pKV79-NDM-9	*bla*_NDM−9_, *bla*_TEM−1B_, *aadA2*, *cmlA1*, *sul1*	3	8.3 × 10⁻^6^	(6.4 × 10⁻^6^ – 1.0 × 10⁻^5^)
pKV102-NDM-9	*bla*_NDM−9_, *bla*_TEM−1B_, *aadA2*, *cmlA1*, *sul1*	3	3.2 × 10⁻^6^	(2.6 × 10⁻^6^ – 3.8 × 10⁻^6^)

### Phylogenetic analysis of ST363 *K. variicola* strains

**Fig. 3:**
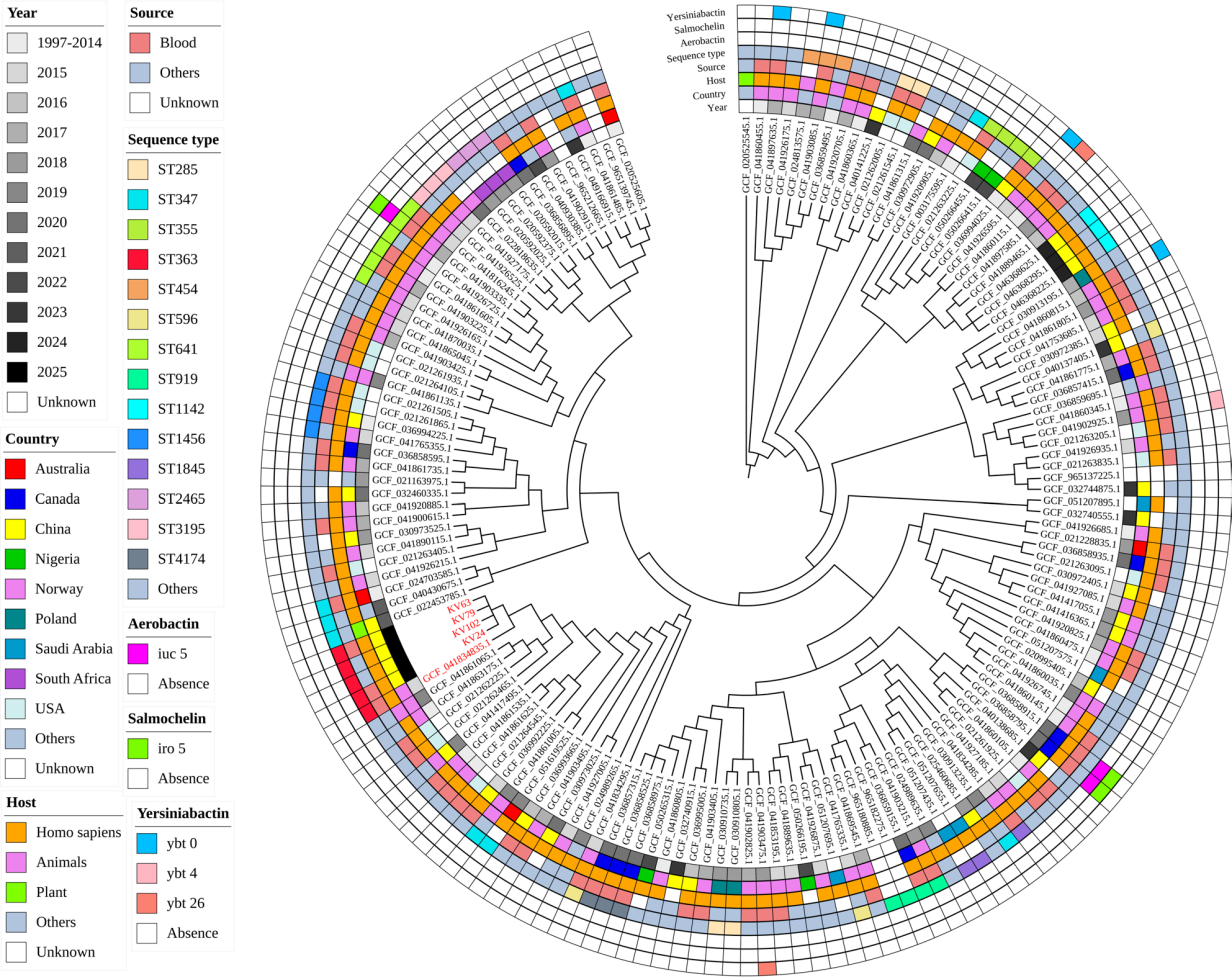
Maximum‑likelihood phylogeny of 160 *K. variicola* genomes inferred from core‑genome SNP variation. The dataset comprises 156 genomes downloaded from NCBI and four ST363‑KL151 clinical isolates sequenced in this work (KV24, KV63, KV79, and KV102; shown in red)

A core-genome SNP phylogeny including 156 publicly available *K. variicola* genomes and the four ST363-KL151 isolates from this study revealed broad geographic and host diversity across the species (Fig. 3). The 160 genomes were derived predominantly from human sources and comprised 103 sequence types. Major hypervirulence-associated loci showed an uneven distribution across the phylogeny. Most *K. variicola* isolates did not carry the principal virulence loci examined, including yersiniabactin (*ybt*), colibactin (*clb*), aerobactin (*iuc*/*iut*), and salmochelin (*iro*). Only seven isolates carried the yersiniabactin gene cluster, including one isolate each belonging to ST1778, ST4315, ST1562, ST4179, ST454, ST725, and ST790, whereas three isolates carried both aerobactin and salmochelin, including two ST5693 isolates and one ST641 isolate. Within this population structure, ST363 was uncommon and formed a distinct monophyletic clade comprising the four study isolates and one additional publicly available genome. The earliest ST363 isolate in the dataset was recovered in Norway in 2015. The four newly sequenced isolates were highly closely related, differing by only 2–12 pairwise SNPs (median, 4). KV24 and KV79 differed by just two SNPs, consistent with very recent shared ancestry and possible local hospital-associated spread.

### Virulence characteristics of ST363‑KL151 *K. variicola* strains

**Fig. 4:**
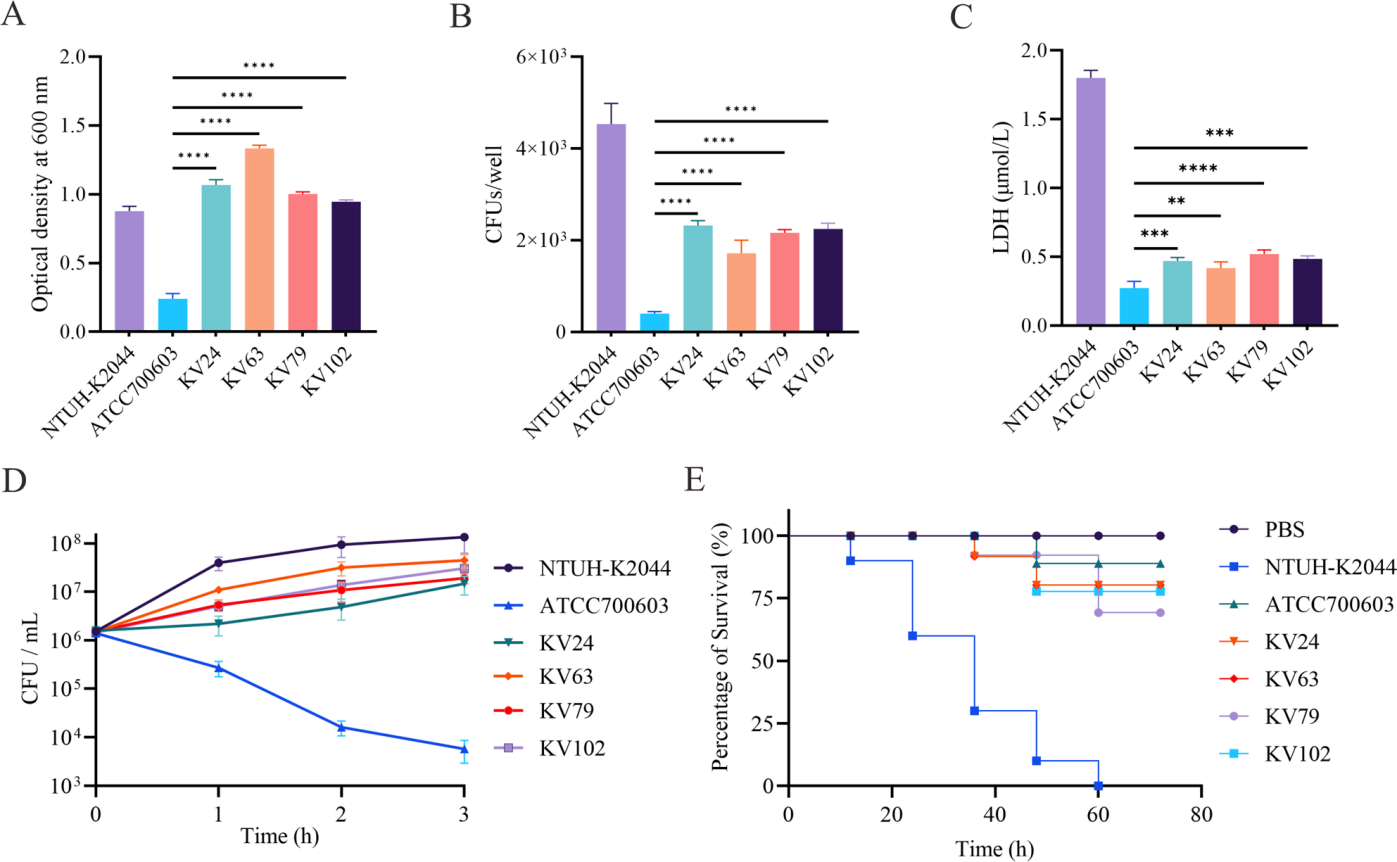
Virulence-associated phenotypes of ST363-KL151 *K. variicola* isolates. **A** Biofilm biomass measured by crystal-violet staining. **B** Invasion of A549 epithelial cells after 30 min of infection. **C** Cytotoxicity determined by LDH release. **D** Serum resistance assessed by viable counts after 3 h incubation in pooled human serum. **E***G. mellonella* survival monitored for 72 h after inoculation

All four ST363-KL151 isolates showed strong biofilm-forming capacity in the crystal violet assay (Fig. 4A), with levels exceeding those of the low-virulence control *K. quasipneumoniae* ATCC 700,603 and comparable to or greater than those of the hypervirulent *K. pneumoniae* strain NTUH-K2044. Among them, KV63 produced the most biofilm. In A549 cell assays, all four isolates exhibited moderate invasion and cytotoxicity (Fig. 4B and C), significantly greater than those of ATCC 700,603 but lower than those of NTUH-K2044. In the serum killing assay, all ST363 isolates showed marked resistance, with viable counts increasing after 3 h of incubation in pooled human serum (Fig. 4D). In the *Galleria mellonella* infection model, however, larvae infected with ST363-KL151 isolates had survival rates above 70% at 72 h, similar to those of the low-virulence control and substantially higher than those of the hypervirulent comparator (Fig. 4E).

## Discussion

*K. variicola*, once regarded mainly as an environmental commensal, is increasingly recognized as a clinically important pathogen causing bloodstream, respiratory, and urinary tract infections [3, 4]. Although only four cases were identified in this study, the range of specimen types and the patients’ severe underlying diseases indicate that *K. variicola* can colonize and infect vulnerable hospitalized individuals. Two patients died, suggesting that severe outcomes may occur, particularly when multidrug resistance is present even in the absence of classical hypervirulence loci. Notably, all four isolates were initially identified as *K. pneumoniae* by MALDI-TOF MS and were reclassified as *K. variicola* only after whole-genome sequencing, which in our study was prompted by their unusual multidrug-resistant phenotype rather than an initial suspicion of *K. variicola*. This finding highlights the difficulty of distinguishing *K. variicola* from *K. pneumoniae* in routine laboratories and suggests that its clinical burden remains underestimated. Contamination was unlikely, as the isolates were recovered from different patients, wards, and specimen types over five months and were all associated with compatible clinical presentations.

Previous studies have shown that *K. variicola* is an underrecognized but clinically significant cause of bloodstream and urinary tract infections [3]. In several reports, isolates initially identified as *K. pneumoniae* were later reassigned to *K. variicola*, often with substantial mortality in bloodstream infection cases [6, 35, 36]. In one multicenter study of 139 bloodstream infections, *K. variicola* accounted for 24.4% of isolates and was associated with a mortality rate of 29.4%, compared with 13.5% for *K. pneumoniae* [6]. Multidrug-resistant *K. variicola* carrying *bla*_NDM‑1_ and *bla*_CTX‑M‑15_ has also been implicated in neonatal sepsis outbreaks [37]. It is also frequently recovered from urinary tract infections, accounting for up to 70% of clinical *K. variicola* isolates in one study [17]. Together, these data indicate that *K. variicola* is a genuine invasive pathogen rather than an incidental isolate.

Genomic studies indicate that *K. variicola* is intrinsically resistant to ampicillin because of the chromosomal β-lactamase LEN, but was historically susceptible to most other agents [17, 18, 38, 39]. This pattern is changing, with increasing reports of multidrug-resistant, ESBL-producing, and carbapenemase-producing *K. variicola* from both clinical and environmental sources [9, 40, 41]. *bla*_NDM_‑producing *K. variicola* has been reported mainly in Asia and Europe [10, 42], suggesting that this species is an underappreciated reservoir of carbapenemase genes within Enterobacterales. Environmental detection on vegetables and in river water further supports its capacity to acquire and disseminate resistance outside hospitals [9, 41, 43]. In the present study, whole-genome sequencing showed that the four *bla*_NDM‑9_‑producing *K. variicola* ST363‑KL151 isolates were clonally related and carried a large IncHI2A plasmid containing most acquired resistance genes, including *bla*_NDM‑9_. Comparative analysis showed that this plasmid was closely related to multidrug-resistant plasmids previously described in *K. pneumoniae* and other Enterobacterales, consistent with a widely distributed IncHI2A resistance plasmid backbone.

Of particular concern is the IS*26*‑flanked *bla*_NDM‑9_ composite transposon accompanied by an downstream mercury resistance operon. This arrangement differs from previously reported *bla*_NDM‑9_ contexts in *K. variicola* and *E. coli* plasmids [9, 44]. A similar mercury-associated *bla*_NDM‑9_ structure has been described in an environmental *K. variicola* isolate from an urban river in South Korea [9], indicating the possibility that such elements may emerge or persist in environmental reservoirs. Together with the plasmid comparison results, this suggests that a conserved multidrug-resistant plasmid scaffold can carry distinct *bla*_NDM−9_-associated resistance regions. This symmetric IS*26*‑bounded organization implies recent IS*26*‑mediated recombination events and may enhance mobilization potential. Conjugation experiments confirmed transfer of carbapenem resistance to *E. coli*, demonstrating the potential of *K. variicola* to contribute directly to horizontal dissemination of *bla*_NDM‑9_. Phylogenetically, the four isolates differed by only 2–12 SNPs and formed a monophyletic clade with a Norwegian strain collected in 2015. Notably, examination of the genome from the isolate collected in Norway showed that it does not harbor a *bla*_NDM−9_-carrying plasmid.

Although the isolates lacked hypervirulence-associated loci such as *rmpA*, *rmpA2*, *iucABCD*, and *iroBCDN*, they retained multiple chromosomally encoded virulence determinants related to adhesion, capsule production, lipopolysaccharide biosynthesis, and iron acquisition. Previous studies have shown that *K. variicola* commonly shares major virulence modules with *K. pneumoniae*, including type 1 and type 3 fimbriae, enterobactin-associated genes, and *wabG*, whereas siderophore loci such as *iucABCD* and *iroBCDN* are uncommon [17, 18]. Our isolates also carried enterobactin-associated genes and other adhesion- or biofilm-related factors, consistent with the observed strong biofilm formation and serum resistance. These findings indicate that *K. variicola* can retain clinically relevant pathogenicity despite lacking canonical hypervirulence plasmids. Phenotypically, the ST363-KL151 isolates showed intermediate virulence, greater than that of the low-virulence *K. quasipneumoniae* reference but lower than that of hypervirulent *K. pneumoniae* NTUH-K2044.

This study is limited by the small number of isolates, which restricts extrapolation to wider epidemiological trends. Additionally, although phenotypic assays provided valuable insights, the absence of mammalian infection models limits direct correlation with human pathogenicity. Future studies using murine infection models will be necessary to validate these findings in vivo. Moreover, environmental sampling within hospital wards might reveal reservoirs or transmission pathways contributing to ST363 persistence. Current publicly available data also suggest that ST363 is a rare sequence type in *K. variicola*, and we found very limited evidence for the ST363-KL151 combination in *Klebsiella*. In our phylogenetic dataset, only one additional publicly available ST363 genome was identified, indicating that the global and clinical prevalence of this lineage remains unclear.

In summary, we characterized the genomic and virulence features of NDM-9-producing *K. variicola* ST363-KL151 isolates from a tertiary hospital in China. Despite lacking classical hypervirulence genes, these strains showed substantial biofilm formation, serum resistance, and transferable carbapenem resistance. These findings underscore the importance of accurate species identification and routine genome-based surveillance to monitor *K. variicola* and limit the spread of plasmid-borne carbapenem resistance.

## Supplementary Information

Supplementary Material 1: Primers used in this study.

## Data Availability

The genome sequences in this study have been submitted to NCBI GenBank under accession number PRJNA1444349.
